# Neurodevelopmental timing and socio-cognitive development in a prosocial cooperatively breeding primate (*Callithrix jacchus*)

**DOI:** 10.1126/sciadv.ado3486

**Published:** 2024-10-30

**Authors:** Paola Cerrito, Eduardo Gascon, Angela C. Roberts, Stephen J. Sawiak, Judith M. Burkart

**Affiliations:** ^1^ Department of Evolutionary Anthropology, University of Zurich, Zürich, Switzerland.; ^2^ Collegium Helveticum, Zürich, Switzerland.; ^3^ Aix Marseille Université, CNRS, INT, Institut Neurosciences de la Timone, Marseille, France.; ^4^ Department of Physiology, Development and Neuroscience, University of Cambridge, Cambridge, UK.; ^5^ Center for the Interdisciplinary Study of Language Evolution (ISLE), University of Zurich, Zürich, Switzerland.

## Abstract

Primate brain development is shaped by inputs received during critical periods. These inputs differ between independent and cooperative breeders: In cooperative breeders, infants interact with multiple caregivers. We study how the neurodevelopmental timing of the cooperatively breeding common marmoset maps onto behavioral milestones. To obtain structure-function co-constructions, we combine behavioral, neuroimaging (anatomical and functional), and neural tracing experiments. We find that brain areas critically involved in observing conspecifics interacting (i) develop in clusters, (ii) have prolonged developmental trajectories, (iii) differentiate during the period of negotiations between immatures and multiple caregivers, and (iv) do not share stronger connectivity than with other regions. Overall, developmental timing of social brain areas correlates with social and behavioral milestones in marmosets and, as in humans, extends into adulthood. This rich social input is likely critical for the emergence of their strong socio-cognitive skills. Because humans are cooperative breeders too, these findings have strong implications for the evolution of human social cognition.

## Teaser

In common marmosets, brain regions implicated in processing social interactions develop slowly until early adulthood.

## INTRODUCTION

Strong social cognition and prosociality are, from a very young age, hallmarks of the human mind compared to the closest living relatives, the nonhuman great apes (*1*). Because of our peculiar life history, characterized by early weaning and extensive allomaternal care starting from very early in infancy, human development is embedded in a world filled with other individuals, including parents, siblings, and other family members. Thus, this is the context in which human toddlers’ strong social cognition and prosociality develops (*2*). It is this same period that is also the most important for the formation of the neural bases of higher-order social, emotional, and communicative functions (*3*). Not unexpectedly then, several independent lines of evidence, spanning neuroscience, pediatrics, primatology, and psychiatry, point to the fundamental role that the relative timing of brain development and social interactions have for the acquisition of social cognition and prosocial behaviors (*4*).

During ontogeny, total brain volume increases until reaching its adult levels. This volumetric increase is the product of gray matter (GM) volume (GMV) increase until a peak value is reached in childhood, after which it decreases concurrently with synaptic pruning and white matter volumetric increase (*5*). In addition, the ontogenetic trajectories of cerebral GM are heterochronous, such that both maximum GMV and GM reduction rate vary across brain regions. The importance of the temporal patterns of brain development in shaping the adult phenotype becomes apparent, for example, in the case of autism spectrum disorder (ASD). Deviations from the normal range of developmental timing of the cortex can profoundly affect socio-cognitive skills and are one of the main factors linked to the occurrence of ASD (*3*). Specifically, several studies have found that early brain overgrowth during the first years of life strongly correlates with ASD [e.g. (*6*)] and a meta-analysis of all published magnetic resonance imaging (MRI) data by 2005 revealed that the period of greatest brain enlargement in autism is during early childhood (*7*), with about a 10% volume increase compared to controls during the first year of life. Hence, individuals affected by ASD present an accelerated early-life brain growth and achieve a final brain volume that is not different from that of controls, but they achieve it earlier than controls. Recent works with human brain organoids has confirmed the accelerated maturation of the cortex in the ASD phenotype, especially interneurons (*8*, *9*). Consequently, given this accelerated early-life brain development, fewer social inputs are available during the period when the GMV reduces to adult size and differentiates via experience-dependent pruning. Accelerated development of functional connectivity between certain brain areas [e.g., amygdala–prefrontal cortex (PFC)] can also be a consequence of early-life stress, which, in turn, can cause adverse physiological conditions such as increased anxiety and cortisol levels (*10*). Unfortunately, so far, nothing is known regarding the impact of changes in brain developmental timing within nonhuman species. That is, we do not know if, within a given nonhuman species, alterations in ontogenetic trajectories of the brain have an impact on the adult behavioral phenotype. However, comparative studies across species with different ontogenetic trajectories and social behaviors can help us shed light on the relationship between the two.

The importance of social inputs occurring during prolonged brain maturation and slow developmental pace has also been highlighted in the context of human evolutionary studies. The remarkable brain growth and development occurring postnatally in humans arguably allows the brain to be influenced by the social environment outside of the uterus to a greater extent than that seen in other great apes (*11*), who are not cooperative breeders (*12*). Hawkes and Finlay (*13*) show that, in addition to weaning our infants earlier than expected (based on allometric scaling with other life-history variables), human neonates have an especially delayed neural development, which is likely correlated with the energetic trade-offs stemming from the large size and high caloric demand of our brain (*14*). In addition, we observe that, in humans, compared to other great apes, myelination is much prolonged and continues well into adulthood (*15*).

Common marmosets (*Callithrix jacchus*) are cooperatively breeding platyrrhine monkeys. Like humans, but unlike other great apes (*12*), they rely on extensive allomaternal care and share many life-history traits (e.g., short interbirth intervals and a hiatus between menarche and first reproduction) with humans (*16*). They also show remarkable prosociality (*4*, *15*) [much more than great apes (*16*)] and strong socio-cognitive abilities, which have been argued to correlate with cooperative breeding (*17*–*20*). However, the neurobiological features underlying the socio-cognitive abilities promoting the prosocial behavior are poorly understood. Moreover, experimental research has shown that, in common marmosets (hereafter marmosets), there is a critical period for the development of social behaviors (*21*), although the relationship between developmental timing of the brain and these early-life social interactions is poorly understood.

Given these similarities with humans, marmosets are becoming an ever-more important model in neuroscience (*22*–*25*) and particularly in research investigating the neurobiological and neurodevelopmental bases of social cognition. As in humans, immature marmosets are surrounded and cared for by multiple caregivers from the first day on. The entire family is typically present during birth, and oxytocin levels increase not only in mothers but also in all group members (*26*). Group members contribute appreciably to carrying the infants and, once infants start eating solid food, frequently share food with them. After a peak provisioning period, adults are increasingly less willing to share food with them (*27*–*29*). During this period, intense and noisy negotiations over food are frequent, with immatures babbling and begging and adults eventually giving in—or not. Intriguingly, when doing so, immatures appear to take into account how willing individual adults are to share and will insist in more and longer attempts with adults who are generally less likely to refuse them. Soon after, immatures have to compete for attention and food not only with their twin sibling but also with the next offspring that are born far before they themselves are independent because, like in humans, marmosets are weaned early and mothers have their next offspring soon after (*30*). By now, the immatures still have not reached puberty; this only happens shortly before yet another set of younger siblings is born. Typically, with these new arrivals, the immatures start to act as helpers themselves and thus face the developmental task of switching from being a recipient of help to becoming a provider of help and prosocial acts (*31*). This is thus the developmental context in which marmosets’ socio-cognitive skills develop.

The goal of this study is to map these behavioral milestones specific to a cooperatively breeding primate to its region-specific brain development to better understand the social interactions in which infants engage during the differentiation period of brain regions selectively implicated in processing social stimuli. Our working hypothesis is that, like in humans, social interactions with several caregivers during this critical period profoundly contribute to the co-construction of the marmoset brain, the maturation of socially related associative areas, and therefore the emergence of prosocial behaviors. For that purpose, we sought to determine if there is a relationship between the temporal profile of the developing marmoset brain and the early-life social interactions that may help explain their sophisticated socio-cognitive skills at adulthood.

To compare the timing of brain development to that of these behavioral milestones and developmental tasks of attaining nutritional independence, we focused on brain regions that, in adult marmosets, are selectively activated by the observation of social interactions between conspecifics but not by multiple but independently behaving marmosets, as identified by Cléry *et al.* (*32*). We tested if these brain regions share similar developmental trajectories based on the developmental patterns of regional GMV. To potentially reveal a coordinated ontogenetic profile underlying the “tuning” of the social brain in marmosets, we then compared these neurodevelopmental patterns to longitudinal data of infant negotiations with caregivers in relation to food (as measured by the frequency of food begging). Last, because it is known that brain regions whose activations correlate with performance on a given task strengthen and get fine-tuned with age (*33*, *34*), we assessed if there is stronger connectedness between areas that develop according to similar developmental trajectories and share similar response to social interaction stimuli.

We thus combined several types of previously published data from marmosets to provide a unified picture of structural brain development alongside the development of social interactions between infants and multiple caregivers necessary to ensure survival (infant provisioning). These included structural MRI (sMRI) data of GM of 53 cortical areas and 16 subcortical nuclei acquired from a developmental cohort (aged 13 to 104 weeks) of 41 male and female marmosets (*35*), functional MRI (fMRI) data mapping the brain areas activated by the observation of social interactions in marmosets (*32*), food sharing interactions in five family groups of marmosets including a total of 26 adults and 14 immatures [from 1 to 60 weeks of age (*27*)], and cellular-resolution data of corticocortical connectivity in marmosets obtained via 143 retrograde tracer injections in 52 young adult marmosets of both sexes (*36*).

Overall, we make the following predictions:

1) P1: Cortical regions that show significantly stronger activation during the observation of social interactions (*32*) share similar structural neurodevelopmental profiles, which are distinct from those regions showing significantly stronger activation during the observation of nonsocial activities.

2) P2: That those same brain regions showing a significantly stronger activation during the observation of social interactions exhibit a protracted development, reaching their adult volume later than the other regions.

3) P3: The developmental trajectory of infant negotiations with caregivers in relation to food (as measured by the frequency of food begging) is more similar to that of brain regions responding more strongly to the observation of social interactions than to the other regions.

4) P4: Functional connectivity is stronger between regions with similar developmental timing and response strength to the observation of either social or nonsocial behaviors and weaker between regions with different developmental timing and response strength.

## RESULTS

Overall, our results support our predictions and show that (i) brain areas that respond significantly more strongly to visual stimuli of social interactions present similar developmental trajectories and thus belong to the same clusters to the exclusion of regions that respond more strongly to visual stimuli of individuals acting alone; (ii) social clusters have an extended developmental trajectory, reaching adult-like volumes later and after a period of plateau; and (iii) the ontogenetic trajectory of food negotiations matches quite well that of “social” brain regions. However, we did not find support for stronger corticocortical connections between areas with stronger responses to visual stimuli of social interactions: Social regions did not appear to form a strongly connected network.

### P1—Do social areas share similar trajectories?

**Fig. 1.**
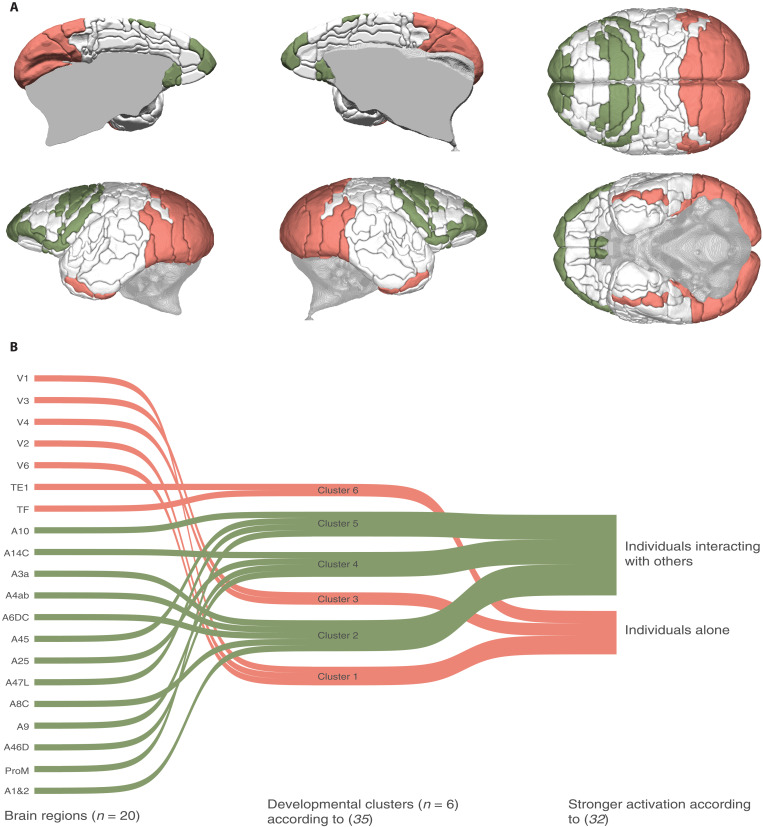
Developmental clusters of the cortical regions analyzed. (**A**) Location of the 20 cortical brain regions that are significantly more active (fMRI data) when observing individuals interacting with conspecifics (green) or individuals acting alone (pink). From the top left moving clockwise: medial views of the left and right hemispheres, superior view, inferior view, and lateral views of the right and left hemispheres. (**B**) Sankey diagram of these same brain regions and their relationship to the six cortical clusters reported in (*35*): the visual and cingulate cluster (1); the somatomotor cluster (2); the auditory-visual cluster (3); the orbitofrontal, dorsolateral, and ventromedial prefrontal cluster (4); the ventrolateral PFC, polar, operculum, and insula cluster (5); and the lateral and inferior temporal lobe cluster (6).

There is a biunivocal correspondence between those brain regions that were differentially activated (as measured via fMRI) by either social interactions or solitary behaviors (Fig. 1A) and neurodevelopmental clusters (Fig. 1B). Specifically, social interactions activated regions fell into three developmental clusters; cortical areas 1 and 2, 3a, 4ab, 6DC, and 8C cluster together; areas 14C, 25, 46D, and 9 belong to another cluster; and areas 10, 45, and 47L and the proisocortical motor region (ProM) belong to yet another cluster. Conversely, the seven cortical areas that responded more strongly to the observation of solitary behaviors fell into the other three of the six developmental clusters: visual areas 1, 2, and 6 form one cluster; visual areas 3 and 4 form another cluster; and temporal area TE1/2 and the ventral temporal lobe form another cluster. As per our prediction, a given developmental cluster contains areas responding more strongly only to one of the two conditions.

### P2—Do social areas have protracted developmental?

**Fig. 2.**
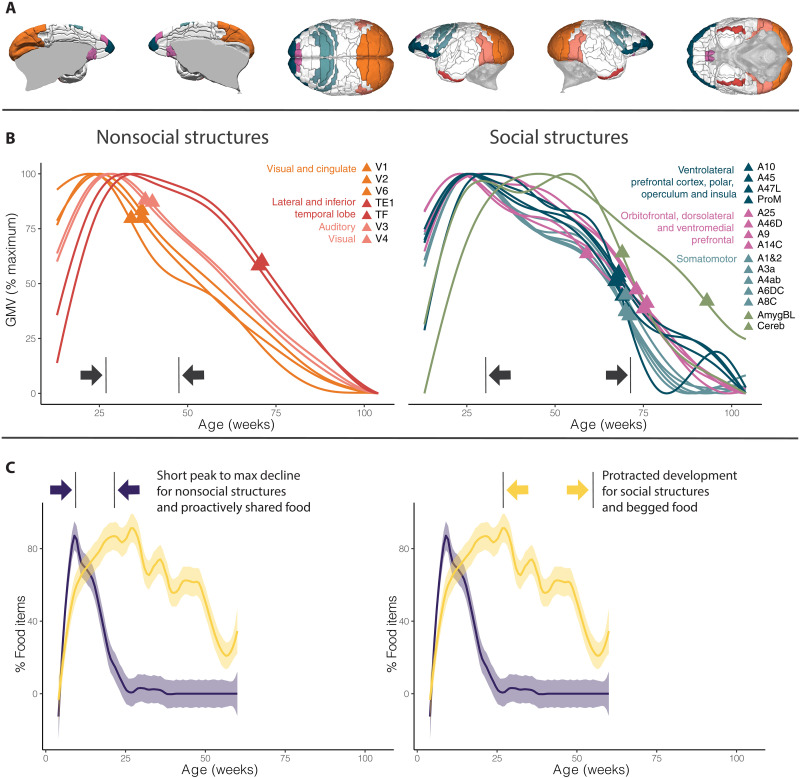
Developmental trajectories of infant-caregiver interactions and of all brain regions analyzed. (**A**) Location of the 20 cortical regions analyzed. Colors represent developmental clusters and match the colors in (B). (**B**) Developmental profiles of the nonsocial (left) and social (right) regions. The triangle is placed on point C (age fastest rate of decline). The colors represent the six developmental clusters of cortical regions, while the subcortical regions (basolateral nucleus of the amygdala and cerebellum) are represented in green. The *x* axis represents time (expressed in weeks from birth), while the *y* axis represents relative volume (absolute values are not reported graphically as there is tremendous variance between regions and would therefore be difficult to visualize all together). (**C**) In blue, the proportion of food that is proactively shared, in yellow the proportion of trials for which infant begging occurs (regardless of the outcome of begging), the shaded areas indicate the 95% confidence interval. The ranges reported in the four plots indicate the time between developmental milestones A (maximum value) and C (maximum rate of decline).

The results of the Bonferroni-corrected pairwise *t* tests for each of the four neurodevelopmental milestones and three ranges (Fig. 2 and Table 1) indicate that the age at the fastest rate of gray volume decline is the only milestone that differs significantly between regions that respond more strongly to visual stimuli of social interaction versus solitary behaviors. Moreover, it is the temporal range between the age at maximum gray volume and the age at the fastest rate of gray volume decline that distinguishes regions with differing activation to the two types of stimuli. The duration of the volumetric “plateau,” measured as the time from the age at maximum volume to the age at which there is the first descending inflection point (figs. S1 and S2), differs significantly (*P* = 0.011) between brain areas that respond more strongly to visual stimuli of social interactions than to those of solitary behavior. The ontogenetic trajectories of the 10 regions that do not differ in response to both stimuli of social interaction and nonsocial activities are shown in fig. S3. These regions are located in the temporal, parietal, and orbitofrontal cortex, which are known to be implicated in both social perception and socioemotional action.

**Table 1.** Differences in developmental timing between regions. Results of Bonferroni-corrected pairwise *t* tests comparing milestone and ranges between brain regions that have significantly stronger activation in response to either social or nonsocial stimuli. Bolded results are significant at *P* < 0.05.

Regions	Variable	*P* value
Social versus nonsocial	A (age at max volume)	0.6168
Social versus all	A (age at max volume)	0.4966
Nonsocial versus all	A (age at max volume)	0.7575
Social versus nonsocial	B (age at first descending inflection point)	0.43
Social versus all	B (age at first descending inflection point)	0.6364
Nonsocial versus all	B (age at first descending inflection point)	0.2415
**Social versus nonsocial**	**C (age at fastest rate of volume decline)**	**0.0015**
**Social versus all**	**C (age at fastest rate of volume decline)**	**0.0008**
**Nonsocial versus all**	**C (age at fastest rate of volume decline)**	**0.0214**
Social versus nonsocial	D (age at last ascending inflection point)	0.46
Social versus all	D (age at last ascending inflection point)	0.1522
Nonsocial versus all	D (age at last ascending inflection point)	0.1366
**Social versus nonsocial**	**A to B (age at max volume to age at first descending inflection point)**	**0.011**
Social versus all	A to B (age at max volume to age at first descending inflection point)	0.188
Nonsocial versus all	A to B (age at max volume to age at first descending inflection point)	0.1064
**Social versus nonsocial**	**A to C (age at max volume to age fastest decline)**	**0.0016**
**Social versus all**	**A to C (age at max volume to age fastest decline)**	**0.0007**
**Nonsocial versus all**	**A to C (age at max volume to age fastest decline)**	**0.0063**
**Social versus nonsocial**	**A to D (age at max volume to age at last ascending inflection point)**	**0.011**
Social versus all	A to D (age at max volume to age at last ascending inflection point)	0.2394
Nonsocial versus all	A to D (age at max volume to age at last ascending inflection point)	0.1065

### P3—Are regional trajectories of social brain areas similar to those of social feeding behaviors?

The results of the comparisons between developmental trajectories of brain regions and food proactively shared or begged support our prediction. The age at fastest decline is much later for the proportion of food begged (58 weeks) than for the proportion of proactively shared food (25 weeks). Moreover, the range between the peak and the age at fastest decline is also larger for the proportion of food begged (31 weeks) than for the proportion of food shared (17 weeks). The ontogenetic trajectory of food begging parallels that of the brain regions with stronger activation in response to visual stimuli of social interactions, while that of proactively shared food parallels that of the brain regions with stronger activation in response to stimuli of solitary behaviors (fig. S4 and Table 2). The ontogenetic trajectories of the two patterns of food provisioning (proactively sharing and begging) parallel those of brain regions (nonsocial and social, respectively) but anticipate them (Fig. 2).

**Table 2.** Ages for the different milestones of neurodevelopment and behaviors. For the ontogenetic trajectories of brain regions, average values and SDs are reported, while for the ontogenetic trajectories of food sharing, the single value is reported (all values are in weeks).

Variable	Average/Value (weeks)	SD
C Social regions	71.3	7
C Nonsocial regions	46.7	16.3
C Proportion begged	58	
C Proportion proactively shared	25	
A to C Social regions	42.2	8.25
A to C Nonsocial regions	19	12.3
A to C Proportion begged	31	
A to C proportion proactively shared	17	

### P4—Do regions with similar GM developmental profiles and fMRI response to stimuli of social interactions have stronger functional connectivity?

Our results (Table 3) indicate that connectivity strength is significantly different (and greater) between those regions that both respond more strongly to nonsocial stimuli, rather than between those responding more strongly to social stimuli. The regions that respond more strongly to nonsocial stimuli are also the ones with faster developmental trajectories, which cluster in groups 1, 3, and 6. The results when the same analysis is performed on regions grouped by developmental cluster, rather than in response to different stimuli, are reported in the Supplementary Materials (fig. S5 and table S1). Models with *Source_to_Target* as the additive term had lower Akaike information criterion (AIC) scores than the corresponding model with *Source_to_Target* as the interaction term, indicating that source to target alters the slope of the relationship between the fraction of extrinsic labeled neurons (FLNe) and age at maximum rate of volumetric decline of the region. The results of the two models with the additive term (tables S1 and S2) indicate that there is a weak correlation between similarity in developmental timing and connectivity strength (for both models, *R*^2^ = 0.2; *P* < 0.0001). We used the AIC (*37*) to compare the two models, and the one having as response variable the absolute difference in the age at maximum rate of volume decline is the best one. Last, in both models, the intercept is significantly different (*P* < 0.0001) only between regions that both respond more strongly to nonsocial stimuli, thus confirming the results of the Wilcoxon rank sum tests (fig. S6).

**Table 3.** Connectivity strength between different regions. Results of the pairwise Wilcoxon rank sum tests (*P* values are corrected for multiple hypothesis testing) on the connectivity strength between brain regions with different activation strengths to different types of stimuli. Values reported as 0 are <0.00000001. NA, not applicable. Bolded results are significant at P < 0.05.

	Neither to neither	Neither to nonsocial	Neither to social	Nonsocial to neither	Nonsocial to nonsocial	Nonsocial to social	Social to neither	Social to nonsocial
**Neither to nonsocial**	1	NA	NA	NA	NA	NA	NA	NA
**Neither to social**	0.44866	1	NA	NA	NA	NA	NA	NA
**Nonsocial to neither**	0.70614	1	1	NA	NA	NA	NA	NA
**Nonsocial to nonsocial**	**0.01302**	**0.00104**	**0.00007**	**0.00072**	NA	NA	NA	NA
**Nonsocial to social**	**0.00001**	**0.00286**	**0.00045**	**0.00503**	**0**	NA	NA	NA
**Social to neither**	1	1	1	1	**0.00049**	**0.00001**	NA	NA
**Social to nonsocial**	**0.00155**	0.44866	0.37016	0.70293	**0**	0.44866	**0.00695**	NA
**Social to social**	1	1	1	1	**0.00093**	**0.00049**	1	0.21406

## DISCUSSION

Our findings reveal that those brain regions more strongly activated when observing social interactions between conspecifics than conspecifics engaging in nonsocial activities have ontogenetic trajectories that differ from those regions that are not more strongly activated (P1). These results thereby indicate that developmental timing and function are correlated in marmosets. Specifically, while the various brain regions do not differ significantly with respect to the age at which they reach their maximum GMV, those involved in processing social interactions maintain the maximum volume significantly longer, before decreasing in size and reaching their adult value (P2). Because GMV decline is a consequence of synaptic pruning and intracortical myelination (*38*), it functionally corresponds to a decrease in developmental plasticity.

Social regions thus remain plastic for longer during ontogeny. Of all the regions analyzed, the one with the latest peak in volumetric decline and therefore the most prolonged plasticity is the basolateral nucleus of the amygdala. This nucleus is implicated in encoding emotional events with reference to their particular sensory-specific features (*39*) and has undergone convergent evolution in its volumetric patterns in cooperatively breeding species including marmosets and humans (*40*). The slowest developing cortical area is 46D. It is located in the dorsolateral PFC, together with areas A10 and A9, which are also slow in developing and respond more strongly to social stimuli. In humans, the dorsolateral PFC has been shown to be implicated in theory of mind (*41*) and in the suppression of selfish behaviors (*42*). The next slowest developing brain region is A14C, located in the ventromedial PFC together with areas 45 and 47L and the ProM. A14C is also known to be heavily implicated in social cognition (*43*), including joint attention (*44*), facial emotion recognition, theory-of-mind ability, and processing self-relevant information in humans (*45*). A longitudinal study in humans affected by ASD has shown that differential activation during a temporal discontinuing task in the ventromedial PFC, including A14C, and cerebellum in these individuals was associated with abnormal functional brain maturation (*46*). In addition, there is a decrease in effective connectivity from the temporal pole (another late-maturing brain region) to the ventromedial PFC and overall lower activation in the latter in ASD (*47*).

Our findings on regional developmental timing in marmosets are similar to those recently described in humans (*5*). Both species have similar temporal ranges, in relation to developmental milestone, in the ages at which the different regions reach their maximum volume (2 to 10 years in humans; roughly corresponding to the 22 to 77 weeks in marmosets) (*48*). The same study on humans also highlighted an important similarity to our findings in marmosets: Primary sensory regions showed earlier post-peak declines, whereas fronto-temporal association cortical areas showed later post-peak declines.

**Fig. 3.**
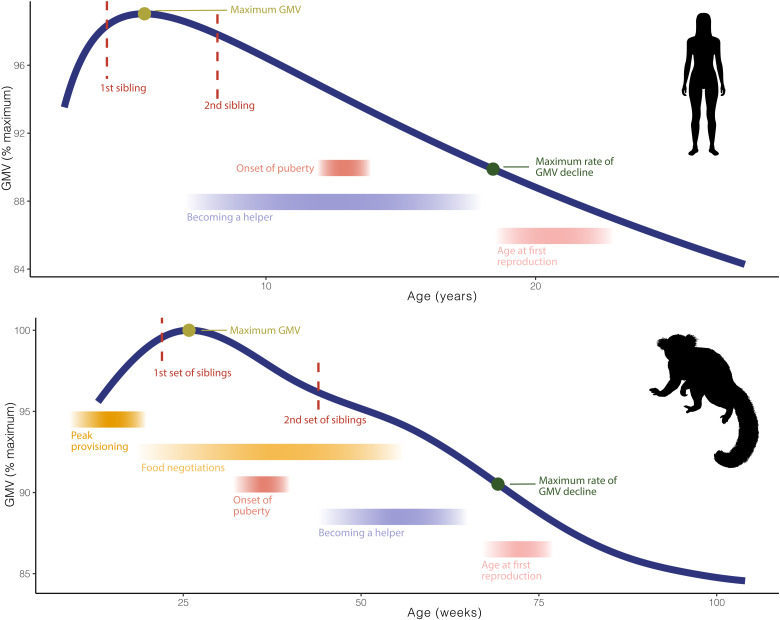
Behavioral milestones and ontogenetic trajectories of GMV in humans and common marmosets. Comparison between cortical GMV trajectories and behavioral and developmental milestones in humans (top) and common marmosets (bottom). Comparative GMV data for humans are from (*5*). Data for behavioral and developmental milestones for humans refer to hunter-gatherer populations and are compiled from several sources (*69*–*71*). Behavioral and developmental data for marmosets are obtained from the following sources: (*27*, *30*).

It is during the period of peak provisioning by nonmaternal family members that the GMV is markedly increasing (Fig. 3), potentially highlighting the fundamental role played by food sharing in providing the nutritional and energetic needs of the developing brain.

The ontogenetic trajectory of regions more strongly activated by the observation of social interactions resembles that of food begging (which is inherently socially interactive) but not of proactively shared food, which does not require active communicative participation from the side of the infant (P3). The proportion of proactively shared food peaks at 8 weeks of age (ranging from week 7 to week 20), a time during which breeders and alloparental caregivers share more than 50% of the food items. Conversely, infant begging peaks at 27 weeks of age, with values remaining high (above 50%) until 50 weeks. Infant begging is a form of interaction and communication that requires an active participation of the infant (unlike proactive sharing), who must learn to engage and elicit care most efficiently from a plurality of individuals (parents and helpers). Anticipating the current motivation and intention of these individuals and adjusting their own solicitation behavior accordingly are important means by which immatures can reach high efficiency in achieving their goal.

Research on humans has shown that the elicitation of care from nonmaternal caregivers entails the mobilization of more regulatory efforts than during the interactions with mothers (*49*). Furthermore, a comparative work on primates (*50*) indicates that, in species practicing alloparental care, there is greater control of the facial musculature and facial expressions, which facilitate nonvocal communication between infants and caregivers. These findings align well with recent works (*51*, *52*) that used fMRI to identify face-selective patches in the marmoset brain and found differential activation in subcortical regions and in the anterior cingulate cortex and lateral PFC while animals viewed socially relevant videos of marmoset faces. This differential activation was observed in many of the brain regions that are also the slowest to reach the age at maximum volume decline, such as the amygdala (102 weeks of age); the cerebellum (92.6 weeks); A32 (82.5 weeks) and A8 (70.1 weeks), which are located in the anterior cingulate; and A47L (68.3 weeks), which is located in the lateral PFC. This combined evidence suggests that not only facial expressions are important for infant-alloparent communication but also the brain regions used to process these expressions have a prolonged plasticity compared to other brain regions (average age at fastest decline across all regions is 65.1 weeks) and that this prolonged ontogenetic trajectory maps onto that of infant-caregiver interactions in relation to food begging.

Limitations of this study are mainly regarding sample sizes. First, while the fMRI data used in our work (*32*) represent the most cutting-edge development and updated data of this type for marmosets, it is nevertheless collected on a small sample of subjects: three adult marmosets (one female and two males). However, other recent works (*52*, *53*) on marmosets using fMRI to differentiate brain regions that are activated by visual stimuli depicting an intact action versus its phase-scrambled version corroborate and replicate the results obtained by Cléry *et al.* (*32*). Further work with an increased number of individuals representing several age groups would provide a valuable validation of our results and the possibility to assess how the structural development and the functional response of brain regions responding more actively to stimuli of social interactions map onto each other. Furthermore, because of slight differences in the parcellation of the cortical areas between the sMRI and the fMRI data (e.g., area 19 in sMRI data and area 19M in fMRI data), it was only possible to match 32 regions. Greater homology in the parcellation used would allow for an increased sample size in regions for which structural and functional data are both available. The connectivity data used for this study, although representing the most extensive anatomical collection, are clearly insufficient. A recent study focusing on nine prefrontal subregions provides an alternative dataset in which, additionally, inputs and outputs have been analyzed separately (*54*). Furthermore, as suggested by accelerated maturation of specific neuronal subsets in ASD (*8*), it would be essential for future experiments to gain access to connectivity patterns of precise cell types in nonhuman primates (NHPs) as previously reported in rodents (*55*). Ultimately, studies such as this one would benefit tremendously from collecting all the different types of data (neuroanatomical, functional, and behavioral) from the same individuals. This is largely limited by the different research questions that are motivating each lab to collect a specific type of data and by the facilities available to each lab. Ideally, future research could consider conducting longitudinal studies in which behavioral, anatomical, and physiological measures are acquired for animals living in as “natural-like” conditions as possible.

Despite the above limitations, several lines of evidence highlight the distinct developmental trajectories of brain regions that display a significantly stronger response to visually presented social interactions compared to those of individuals acting alone. Specifically, regions implicated in the evaluation of social interactions have prolonged neurodevelopmental time periods. Furthermore, we show that this same neurodevelopmental trajectory mirrors, with a temporal shift of about 20 weeks, that of infant food begging, a form of care elicitation and infant-adult interaction that is necessary to ensure infant survival in cooperatively breeding species. More broadly, our results are in line with a recent work (*56*) showing that, in marmosets, the critical period for the acquisition of cognitive control is between 39 and 65 weeks of age, falling within the temporal period from the age at maximum total GMV and the age at fastest GMV decline (Fig. 3). This temporal range is also the one during which individuals transition from being a recipient of help to becoming a helper (*31*), which arguably requires inhibitory control (i.e., to share rather than consume food items) (*57*). Our results are further supported by previous findings (*58*) showing that, in marmosets, family size has an effect on the age at which brain regions mature, with individuals belonging to larger families (and therefore having to integrate a larger number of different social interactions) having protracted neurodevelopment compared to those living in smaller families. This could be explained by the need to become helpers earlier on, if no older sibling are present; however, further research taking into account not only family size but also social roles within the family group is necessary to better understand the mechanism at play.

Last, our current work underscores the notable similarity in the relationship between brain developmental patterns and behavioral milestones in marmosets and humans (Fig. 3). In both species, perhaps because of the short interbirth intervals, immatures become helpers and become physiologically capable of reproduction while GMVs are still drastically changing and even before GMV reaches its maximum rate of decline. In addition, both species experience a hiatus between the onset of puberty and the age at which first reproduction actually occurs. This is likely because prior social experience with being a helper is necessary to carry out a successful reproductive event. Reproduction in callitrichids often fails if individuals have not been helpers first (*59*).

Phenotypic convergences between distantly related species as a consequence of changes in developmental timing are all but unexpected. Across mammals, both allomaternal behavior and developmental timing are phylogenetically labile compared to most other traits (*60*, *61*). Hence, heterochrony (i.e., a difference in the timing, rate, or duration of a developmental process) can likely cause the phenotypic convergence observed in marmosets and humans, two species whose evolutionary trajectories diverged ~42 million years ago (*62*) but that, in many life-history aspects, resemble each other more than each one resembles its sister taxon. Moreover, it is known that small differences in timing can result in large phenotypic changes, such that, for example, heterochronic divergence can cause pedamorphosis (i.e., the retention of an ancestral juvenile trait into adulthood) due to neoteny (*63*). Hence, changes in the timing of life-history events, in relation to the relatively phylogenetically conserved neurodevelopmental schedule (*64*), can cause convergence in the adult phenotype. Future comparative studies of the neurodevelopmental and behavioral trajectories of independently breeding, closely related taxa are the next fundamental step to effectively understand how the interplay between anatomical and behavioral timing shapes the prosocial behavior. In addition, data including nonprimate cooperative breeders and independently breeding taxa have the potential to further corroborate the evolutionary convergence between protracted neurodevelopment, breeding system, and social behaviors.

While life-history and cognitive convergences between humans and marmosets have been abundantly documented, here we provide the first comparative evidence of what is potentially the developmental trajectory underlying the emergence of the adult phenotype [i.e., we address the mechanistic and ontogenetic aspect of the question, sensu Tinbergen (*65*)]. We argue that it is a delayed and prolonged brain development occurring during a time in which infants actively engage in social interactions and negotiations that allows for the emergence of prosociality: The brain needs time with alloparents and selective pressures to interact with them to be tuned toward prosociality. Our results further support the notion that marmoset is an appropriate model to investigate the neurobiological underpinnings of human prosociality.

## MATERIALS AND METHODS

Animal experiment procedures were in accordance with the UK Animals (Scientific Procedures) Act 1986 under license PPL70/7618 and approved by the University of Cambridge Animal Welfare and Ethical Review Board.

### Experimental design

#### Cortical and subcortical structural developmental data

The data were collected in 41 male and female animals aged between 13 and 104 weeks, with each animal scanned at least twice using a Bruker PharmaScan 47/16 MRI system (Bruker Inc., Ettlingen, Germany). Temporal milestones describing the structural developmental trajectories of the 69 brain regions considered have already been published (*35*). The data include 53 cortical areas and 16 subcortical nuclei. MRI images were acquired using a Bruker PharmaScan 47/16 MRI system (Bruker Inc., Ettlingen, Germany) with a 4.7-T magnet. Structural images were acquired using a rapid acquisition with relaxation enhancement sequence (parameters: TR/TEeff 11 750/23.5 ms, 125 slices of 250 μm in thickness, and echo train length 4 and 3 averages) in 21 min and 44 s. The field of view was 64 mm by 50 mm, yielding an isotropic resolution of 250 μm. A cortical atlas based on (*66*) and with further subcortical regions delineated based on (*67*) was used to extract regional volumes from each scan at each time point. This was done using image registration to warp labels from the template based on the warps needed to match individual MRI images to the template using the DARTEL algorithm (*68*). The volumes were used to calculate growth trajectories for each region (data S1) from infancy to adulthood using a cubic spline model allowing for individual offsets using an additive mixture model (Matlab, MathWorks Inc.). Ten knots covering 3 to 24 months were used (*35*). In the present work, we accessed nonpublished, GM region-specific volumetric data, compiled across individuals using cubic spline models, following (*35*). Because both hemispheric asymmetry and sexual dimorphism were tested for and excluded (*35*), we use pooled male and female right and left hemisphere values.

#### fMRI data

These data were obtained from a published source (*32*) and were acquired on male and female adult awake marmosets. Each animal was recorded while presented with video stimuli of social interactions (playing, grooming, etc.) and nonsocial behaviors (eating, foraging, etc.) as well as the scrambled versions of the same videos (four conditions in total). For each monkey, all four conditions were analyzed and compared, revealing the brain regions that show significantly stronger activation while observing the social condition comparing to all other three conditions (*32*). These areas have been coded as “social” in our dataset. The areas showing instead a significantly stronger activation during the observation of nonsocial behaviors have been coded as “nonsocial” and all others as “neither” (data S1).

#### Provisioning data

Infant provisioning patterns were recorded in five family groups, including a total of 14 immatures (aged 1 to 60 weeks) and 26 adults, representing both male and female breeders and male and female helpers (older siblings) (*27*). Once a food item was given to an individual, it was recorded if each food item was shared with the infant proactively, was shared with the infant in response to infant’s begging, or was refused to a begging infant. Hence, sharing could be either proactive, facilitated, or resisted, while the response to begging could be either refusing or sharing. The total proportion of food proactively shared and begged was then compiled across infants and family groups for each point in time, providing a developmental trajectory of both (data S2). The trajectories of proactive, facilitated, and resisted sharing are very similar among each other (*27*). Conversely, the proportion of refused food increased as the one of shared food decreased. The trajectory of food begging reflects the complex negotiation dynamic that goes from helplessly receiving to increasingly more interacting with caregivers to elicit a desired outcome (food). These interactions and negotiations with multiple caregivers are a behavioral peculiarity of cooperatively breeding animals (such as humans and marmosets), which entail infant provisioning by a variety of individuals as a consequence also of prolonged post-weaning dependence (*16*).

#### Corticocortical connectivity data

These data have been published in an open-access form (*36*) and are available on the following web platform: https://marmosetbrain.org. We used the full FLNe connectivity matrix, which was obtained via 143 retrograde tracer injections in 52 young adult marmosets of both sexes. The matrix reports the results of injections centered in 55 target areas of the marmoset cortex, including subdivisions of prefrontal, premotor, superior temporal, parietal, and occipital complexes. The data represent the weighed and directed connectivity matrix based on the results of injections of monosynaptic retrograde fluorescent tracer injections. The rows and columns of the matrix represent individual cortical areas. The targets are the injected areas, while the sources indicate the areas in which the projections originate. The intrinsic connections are excluded from our analysis.

### Statistical analysis

#### P1—Do social areas share similar trajectories?

To obtain a finite number of meaningfully different developmental trajectories of the cortical areas, we followed (*58*) and identified six clusters: the visual and cingulate cluster; the somatomotor cluster; the auditory-visual cluster; the orbitofrontal, dorsolateral and ventromedial prefrontal cluster; the ventrolateral PFC, polar, operculum, and insula cluster; and the lateral and inferior temporal lobe cluster. Increasing cluster numbers resulted in the further subdivision of the PFC, which is the area presenting the largest heterogeneity of developmental trajectories. We then matched the sMRI and fMRI data, which resulted in 32 regions for which we had both types of data (data S1). Of these 32 regions, 15 respond more strongly to social interaction stimuli, 7 respond to stimuli of individuals acting alone, and 10 respond with nonsignificantly different strength to both types of stimuli. The clusterization of regions based on their developmental trajectory, combined with the matching of the sMRI and fMRI data, allowed us to test if regions with similar response to social or nonsocial visual stimuli present similar developmental trajectories (i.e., belong to same developmental clusters) and therefore if developmental timing and socio-cognitive function are correlated. Subcortical nuclei are excluded from this clusterization.

#### P2—Do social areas have protracted development?

To quantify and compare the developmental timing of the different regions, we defined four different milestones: (A) the age at maximum volume, (B) the age at the first descending inflection point (the first local maxima of the first derivative), (C) the age at maximum rate of volume decline, and (D) the age at the last ascending inflection point (the last local minima of the first derivative). Three of these milestones (A, B, and C) are the standard ones that have been previously used (*35*), while we decided to introduce a novel one (D) to capture the decrease in rate of decline visible toward the end of the trajectories. For a visual representation and example of the four milestones, see figs. S1 and S2. Using these four milestones, we defined three temporal ranges: the age at maximum volume to the age at first descending inflection point (A to B), the age at maximum volume to the age at fastest decline (A to C), and the age at maximum volume to the age at the last ascending inflection point (A to D). Last, we proceeded to compute Bonferroni-corrected pairwise *t* test for each of the four milestones and three ranges to assess if they were significantly different between social and nonsocial regions.

#### P3—Are regional trajectories of social brain areas similar to social feeding behaviors?

On the basis of the results of the analyses carried out to verify P2, we selected the milestones and ranges that distinguish the developmental trajectories of social regions from nonsocial ones and computed them also for the developmental trajectories of proactive food sharing and food begging. We then compared the values (in weeks) obtained for the provisioning data to those of the neurodevelopmental trajectories to test our prediction that interindividual negotiations (such as those occurring when infants beg caregivers for food) follow a similar developmental pattern to that of social brain regions.

#### P4—Do regions with similar GM developmental profiles and fMRI response to stimuli of social interactions have stronger functional connectivity?

First, we matched the corticocortical connectivity data with the GM neurodevelopmental data, which resulted in a dataset of 1400 connections encompassing 28 target and 51 source regions (data S3). We also matched the connectivity data with the fMRI data and included only the connections for which fMRI data are present for both the target and the source. This resulted in 494 connections between 19 target and 27 source regions (data S4). To test if connection strength differs between regions with similar response to social or nonsocial visual stimuli, we performed Wilcoxon rank sum tests and adjusted the results for multiple hypothesis testing. We used nonparametric tests because of the non-normal distribution of the data. We also performed the same tests on the data grouped according to the six developmental clusters. To test if there is a correlation between developmental timing and connection strength across regions, for each connection, we computed the average age at peak rate of volume decline between the source and target region and the absolute difference in the age at peak rate of volume decline between the source and target region. We then built four models with FLNe as the response variable and either the average age at peak rate of volume decline (avg_age_max_rate) or absolute difference in age at peak rate of volume decline (d_age_max_rate) as explanatory variables, and fMRI response type in source and target regions as either the (i) additive variable: lm1 = *FLNe ~ avg_age_max_rate + Source_to_Target* and lm2 = *FLNe ~ d_age_max_rate + Source_to_Target* or (ii) interaction term: lm3 = FLNe ~ avg_age_max_rate * Source_to_Target and lm2 = FLNe ~ d_age_max_rate * Source_to_Target. We compared each pair of models (with *Source_to_Target* as either the additive or interaction term) using the AIC (*37*).
