# Supplementary material for: Neurodevelopmental timing and socio-cognitive development in a prosocial cooperatively breeding primate (Callithrix jacchus)
Source: Sci Adv. 2024 Oct 30;10(44):eado3486. doi: 10.1126/sciadv.ado3486 (PMC11804945; doi:10.1126/sciadv.ado3486)
Supplement: Supplementary file 1 — Figs. S1 to S6 Tables S1 and S2 Legends for data S1 to S4 [file sciadv.ado3486_sm.pdf]

Science Advances
AAAS

# Supplementary Materials for

## Neurodevelopmental timing and socio-cognitive development in a prosocial cooperatively breeding primate (*Callithrix jacchus*)

Paola Cerrito *et al.*

Corresponding author: Paola Cerrito, paola.cerrito@uzh.ch; Judith M. Burkart, judith.burkart@iea.uzh.ch

*Sci. Adv.* **10**, eado3486 (2024)
DOI: 10.1126/sciadv.ado3486

**The PDF file includes:**

Figs. S1 to S6
Tables S1 and S2
Legends for data S1 to S4

**Other Supplementary Material for this manuscript includes the following:**

Data S1 to S4

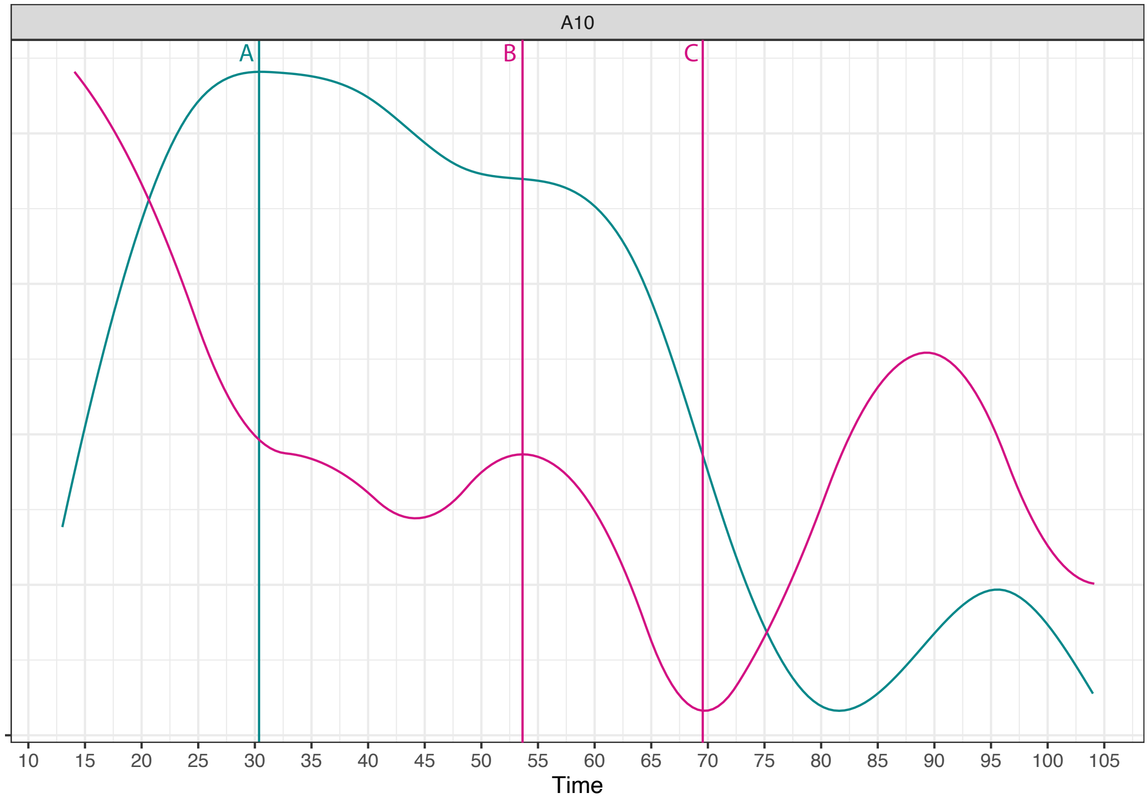


**Fig. S1.**

Definition of developmental milestones A, B and C exemplified on region A10. Green: volume changes over time. Pink: First derivative of green curve. Y-axis is different for the pink and green curves, but not relevant here. A = age at maximum volume; B = Age at first descending inflexion point (the first local maxima of the first derivative); C = age at maximum rate of decline (global minima of the first derivative).

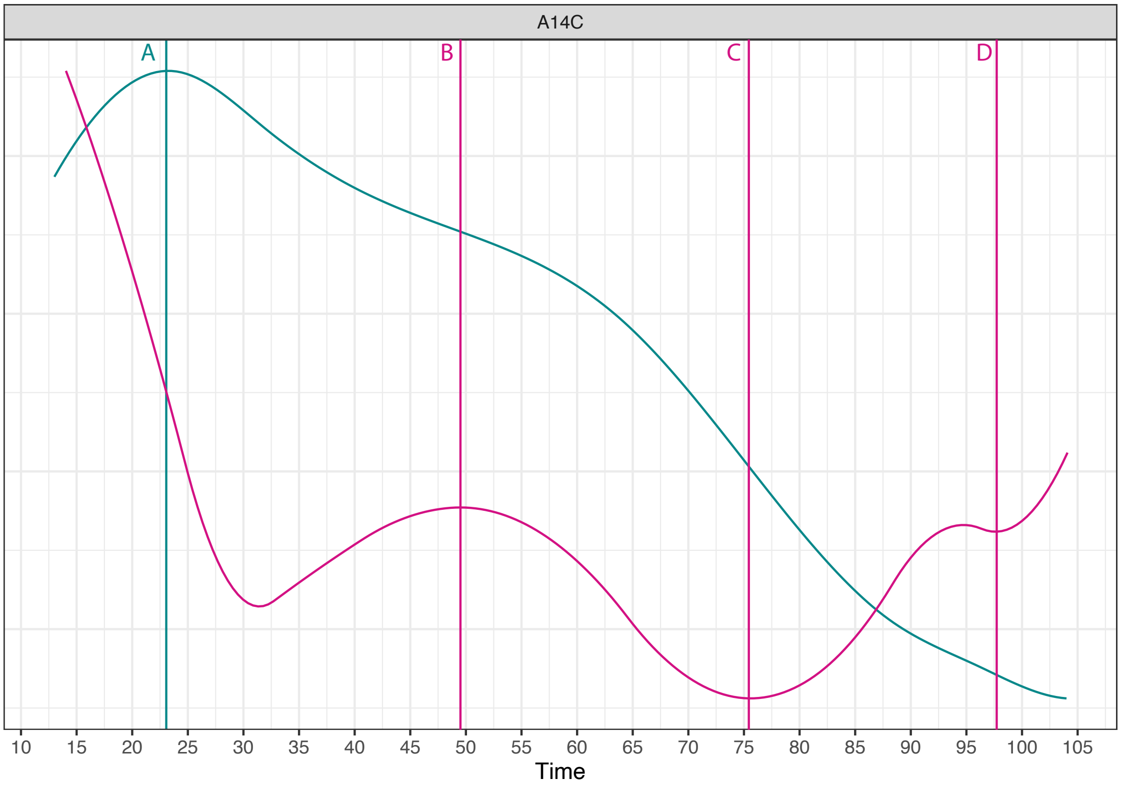


**Fig. S2.**

Definition of developmental milestones A, B, C and D exemplified on region A14C. Green: volume changes over time. Pink: First derivative of green curve. Y-axis is different for the pink and green curves, but not relevant here. A = age at maximum volume; B = Age at first descending inflexion point (the first local maxima of the first derivative); C = age at maximum rate of decline (global minima of the first derivative); D = age at last local minima of the first derivative.

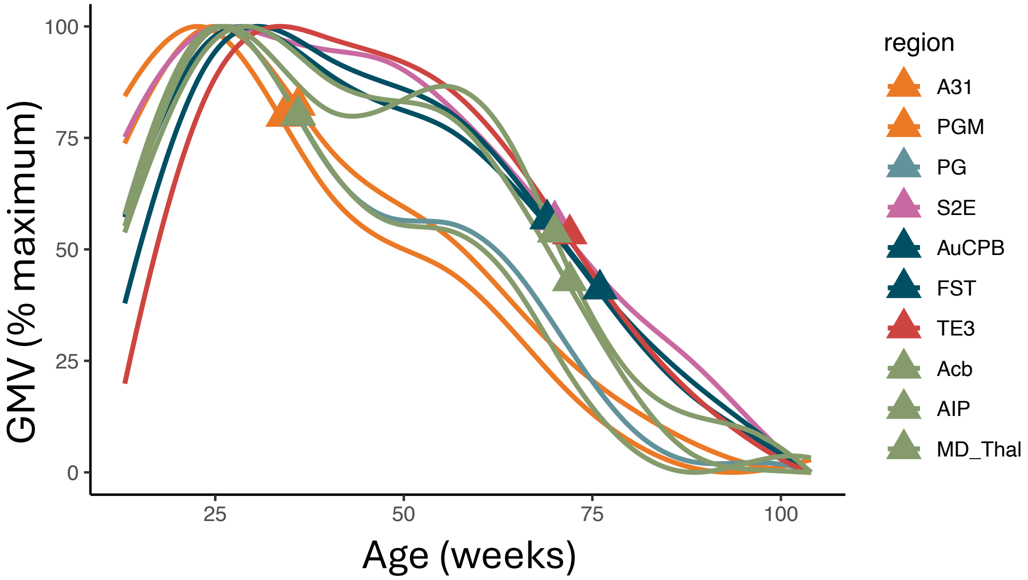


**Fig. S3.**

Developmental profiles of the regions in which the fMRI response is not significantly different between "social" and "non-social" stimuli. The triangle is placed on point C (age fastest rate of volumetric decline). The colors (same as in Fig. 2) represent the six developmental clusters of cortical regions (orange: visual and cingulate; light blue: somatomotor; pink: orbitofrontal, dorsolateral and ventromedial prefrontal; dark blue: ventrolateral prefrontal cortex, polar, operculum and insula; red: lateral and inferior temporal lobe), while the sub-cortical regions (basolateral nucleus of the amygdala and cerebellum) are represented in green. The x-axis represents time (expressed in weeks from birth), while the y-axis represents relative volume (absolute values are not reported graphically as there is tremendous variance between regions and would therefore be difficult to visualize all together). These regions with non-significantly different fMRI response to the two types of stimuli present developmental profiles that are similar to both categories of regions shown in Fig. 2. This likely indicates that, while regions that respond more strongly to stimuli of social interactions have prolonged development, having a prolonged development is not a characteristic exclusive to those regions.

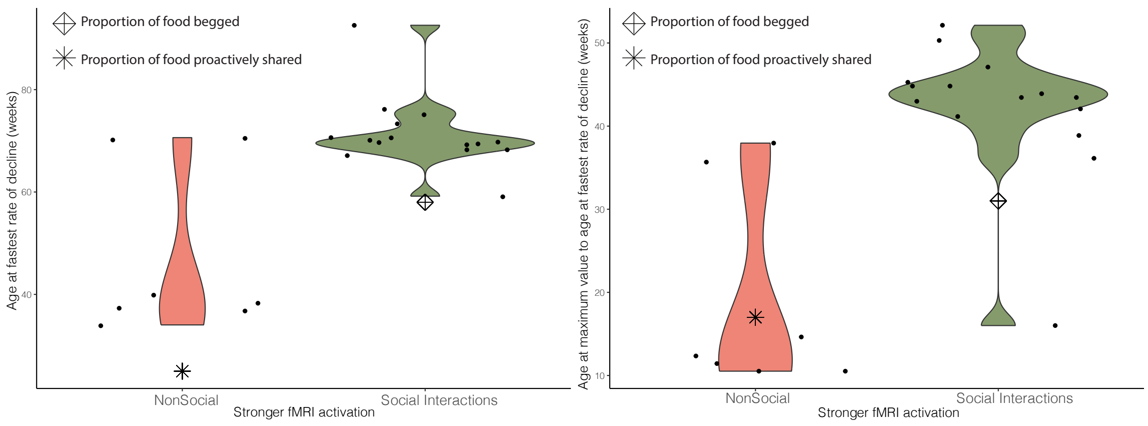


**Fig. S4.**

For social regions (i.e. those activated when observing social interactions, green), the fastest decline of gray matter volume occurs later than for non-social regions (left), and the plateau from the maximum value to the fastest decline is longer (right). These same milestones and ranges are also shown for the ontogenetic trajectories of proactive food sharing (star) and food begging and negotiation (diamond). Individual dots represent individual brain regions.

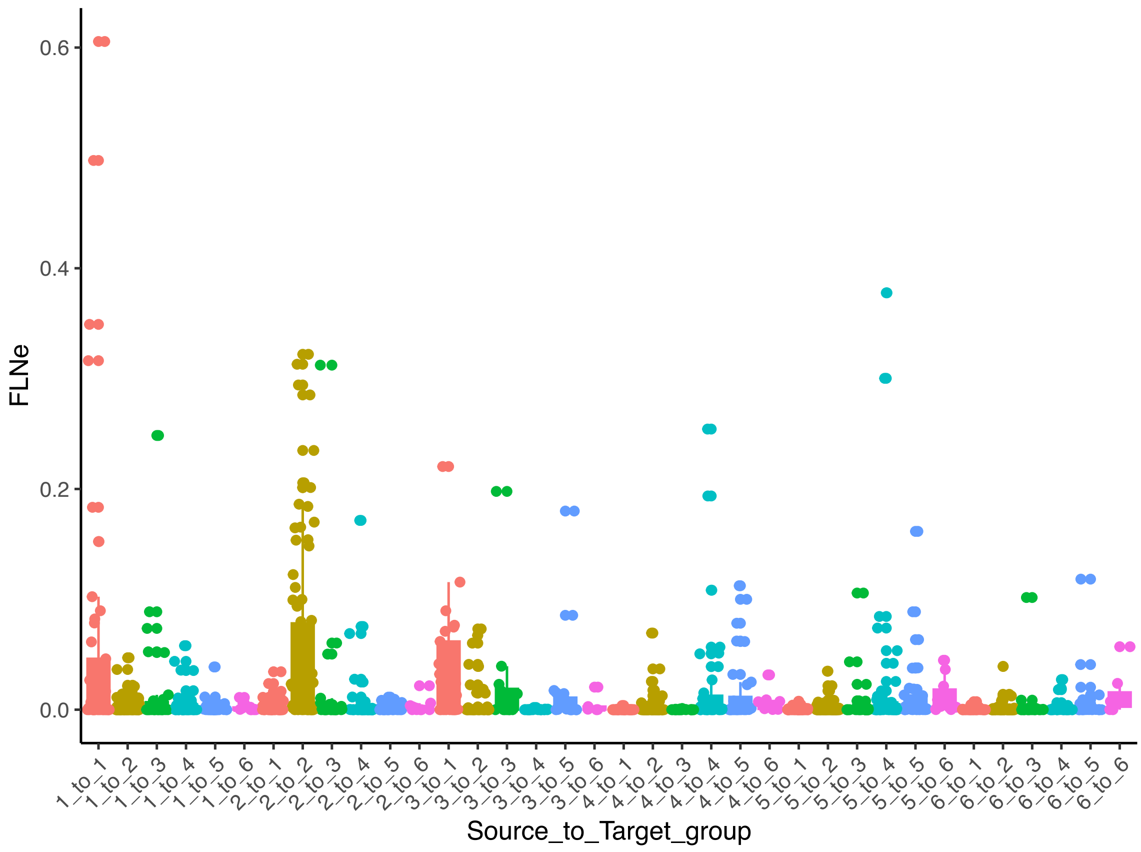


**Fig. S5.**

Fraction of extrinsic labeled neurons (FLN2) colored according to the developmental cluster of the target regions: the visual and cingulate cluster (1); the somatomotor cluster (2); the auditory-visual cluster (3); the orbitofrontal, dorsolateral and ventromedial prefrontal cluster (4); the ventrolateral prefrontal cortex (PFC), polar, operculum and insula cluster (5); the lateral and inferior temporal lobe cluster (6). For the names of each region pertaining to each cluster see Figure 1 and Data S1.

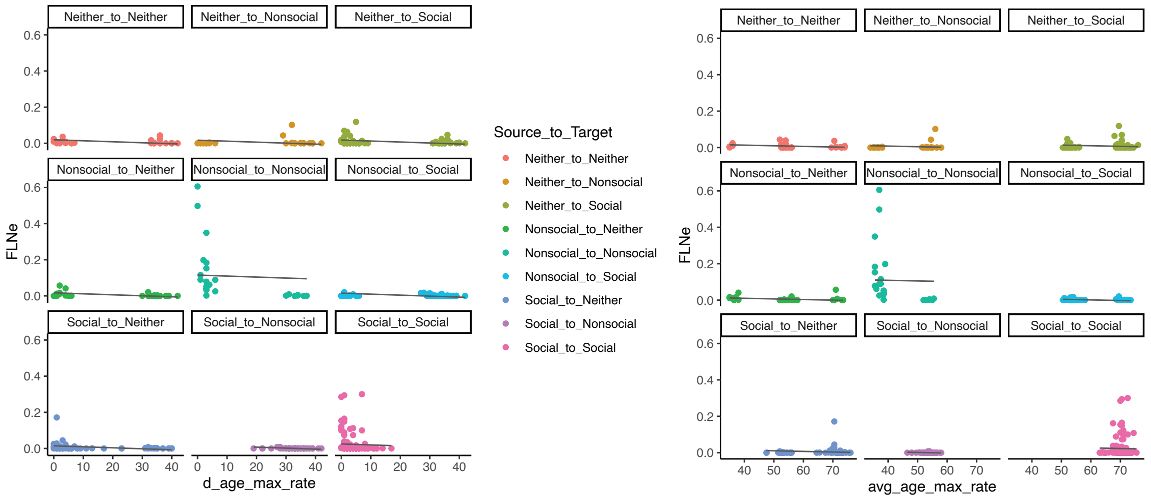


**Fig. S6.**

Plots showing the correlation between fraction of extrinsic labeled neurons (FLNe) and two different measures of similarity in developmental timing (expressed in weeks). Left: absolute difference in the age at which the maximum rate of GM volumetric decline is reached. Right: average age at which the maximum rate of GM volumetric decline is reached. The regression lines are those for FLNe ~ d_age_max_rate + Source_to_Target (left) and FLNe ~ avg_age_max_rate + Source_to_Target (right). In both models, the intercepts are significantly different only for non-social to non-social regions. The overall R-squared is 0.2 for both models.

**Table S1.**

Results of the *FLNe ~ d_age_max_rate + Source_to_Target* model.

| term | estimate | std.error | statistic | p.value |
|---|---|---|---|---|
| **(Intercept)** | **0.019** | **0.009** | **2.102** | **0.036** |
| **Connectivity_S$d_age_max_rate** | **-0.001** | **0.000** | **-3.338** | **0.001** |
| Connectivity_S$Source_to_TargetNeither_to_Nonsocial | -0.002 | 0.012 | -0.184 | 0.854 |
| Connectivity_S$Source_to_TargetNeither_to_Social | -0.001 | 0.010 | -0.140 | 0.889 |
| Connectivity_S$Source_to_TargetNonsocial_to_Neither | -0.002 | 0.012 | -0.214 | 0.831 |
| **Connectivity_S$Source_to_TargetNonsocial_to_Nonsocial** | **0.096** | **0.013** | **7.461** | **0.000** |
| Connectivity_S$Source_to_TargetNonsocial_to_Social | -0.004 | 0.010 | -0.433 | 0.665 |
| Connectivity_S$Source_to_TargetSocial_to_Neither | -0.005 | 0.010 | -0.461 | 0.645 |
| Connectivity_S$Source_to_TargetSocial_to_Nonsocial | -0.001 | 0.011 | -0.096 | 0.924 |
| Connectivity_S$Source_to_TargetSocial_to_Social | 0.006 | 0.010 | 0.574 | 0.566 |

**Table S2.**

Results of the *FLNe ~ avg_age_max_rate + Source_to_Target* model.

| term | estimate | std.error | statistic | p.value |
|---|---|---|---|---|
| **(Intercept)** | **0.027** | **0.018** | **1.500** | **0.134** |
| Connectivity_S$avg_age_max_rate | 0.000 | 0.000 | -1.240 | 0.215 |
| Connectivity_S$Source_to_TargetNeither_to_Nonsocial | -0.005 | 0.013 | -0.405 | 0.686 |
| Connectivity_S$Source_to_TargetNeither_to_Social | 0.004 | 0.010 | 0.350 | 0.727 |
| Connectivity_S$Source_to_TargetNonsocial_to_Neither | -0.003 | 0.012 | -0.255 | 0.799 |
| **Connectivity_S$Source_to_TargetNonsocial_to_Nonsocial** | **0.096** | **0.013** | **7.123** | **0.000** |
| Connectivity_S$Source_to_TargetNonsocial_to_Social | -0.005 | 0.010 | -0.493 | 0.622 |
| Connectivity_S$Source_to_TargetSocial_to_Neither | 0.001 | 0.011 | 0.087 | 0.930 |
| Connectivity_S$Source_to_TargetSocial_to_Nonsocial | -0.008 | 0.011 | -0.750 | 0.454 |
| Connectivity_S$Source_to_TargetSocial_to_Social | 0.020 | 0.010 | 1.972 | 0.049 |

**Data S1 (separate file).** List of brain regions included in Sawiak et al. (***35***) and Cléry et al (***32***). For each brain region, if available, we also report the developmental cluster and fMRI response to the different conditions analyzed.

**Data S2 (separate file).** Provisioning and infant begging ontogenetic data (from weeks 4 to 60 of age). As reported in Guerriero-Martins et al. (***27***), values for each category (proactively shared, facilitated, resisted, begged, shared, refused) are reported as percentages. Shared is the sum of proactive, facilitated and resisted; begged is the sum of facilitated, resisted and refused.

**Data S3 (separate file).** Corticocortical connectivity strength (***36***) for regions for which GMV neurodevelopmental data is also present in both target and source regions. This results a dataset of 1400 connections encompassing 28 target and 51 source regions. For each of the two regions in each connection we also report: the developmental cluster (see methods section of main manuscript file), the age at maximum rate of volume decline and the age at maximum volume. Finally, we also report the difference (between the regions forming each connection) in age at maximum volume and age at maximum rate of volume decline.

**Data S4 (separate file).** Corticocortical connectivity strength (***36***) for regions for which fMRI response data to the different types of social stimuli is also present in both target and source regions. This results a dataset of 494 connections between 19 target and 27 source regions. For each of the two regions in each connection we also report: the fMRI response type (see methods section of main manuscript file), the developmental cluster, the age at maximum rate of volume decline and the age at maximum volume. Finally, we also report the difference (between the regions forming each connection) in age at maximum volume and age at maximum rate of volume decline.
